# The Burden of Non-Alcoholic Fatty Liver Disease in Adolescents with Polycystic Ovary Syndrome: A Case–Control Study

**DOI:** 10.3390/jcm12020557

**Published:** 2023-01-10

**Authors:** Aikaterini Giannouli, Vasiliki Efthymiou, Marianna Konidari, Iliana Mani, Leon Aravantinos, Spyridon P. Dourakis, Aristeidis Antoniou, Efthymios Deligeoroglou, Flora Bacopoulou

**Affiliations:** 1 Center for Adolescent Medicine and UNESCO Chair in Adolescent Health Care, First Department of Pediatrics, Medical School, National and Kapodistrian University of Athens, Aghia Sophia Children’s Hospital, 1 Thivon Str., 11527 Athens, Greece; 2 First Department of Radiology, Medical School, National and Kapodistrian University of Athens, Aretaieio Hospital, 76 Vassilisis Sofias Ave., 11528 Athens, Greece; 3 Research Laboratory, Second Department of Internal Medicine, Medical School, National and Kapodistrian University of Athens, Hippokratio Hospital, 11527 Athens, Greece; 4 Second Department of Obstetrics and Gynecology, Medical School, National and Kapodistrian University of Athens, Aretaieio Hospital, 76 Vassilisis Sofias Ave., 11528 Athens, Greece; 5 Department of Pediatric & Adolescent Gynecology, Mitera Children’s Hospital, Erythrou Stavrou, 15123 Athens, Greece

**Keywords:** adolescent, PCOS, polycystic ovary syndrome, fatty liver, NAFLD, MAFLD, non-alcoholic fatty liver disease, liver steatosis, insulin resistance, obesity

## Abstract

The aim of this case–control study was to assess the burden of non-alcoholic fatty liver disease (NAFLD) in adolescents with polycystic ovary syndrome (PCOS) and its associations with insulin resistance, hyperandrogenism, and other metabolic characteristics of the syndrome. A total of 87 Caucasian adolescent girls (47 with PCOS and 40 controls), aged 12.3–20.4 years, underwent blood sampling for glucose metabolism, hormonal and lipid profile, gynecological and liver ultrasound, and liver elastography. Indices of insulin resistance, liver steatosis, and liver fibrosis were calculated. NAFLD diagnosed by ultrasound was more prevalent in adolescents with PCOS than controls (22.7% vs. 6.1%, *p* = 0.046), and was also verified by liver steatosis indices. The latter was not apparent for hepatic fibrosis, as assessed by Fibroscan^®^ and calculated indices. The homeostatic model assessment for insulin resistance (HOMA-IR) was found to predict NAFLD diagnosis by the liver fat score (LFS) index (β = 0.709, *p* = 0.002). Adolescents with PCOS and high free androgen index (FAI) presented worse NAFLD than those adolescents with PCOS and lower FAI. In addition, adolescents with PCOS and concurrent NAFLD had worse insulin sensitivity indices (HOMA-IR, QUICKI, and glucose to insulin ratio) than adolescents with PCOS alone. Adolescent insulin resistance could be considered a confounder of the association between PCOS and NAFLD.

## 1. Introduction

Polycystic ovary syndrome (PCOS) is a common multifactorial disorder affecting women of reproductive age. The diagnosis of PCOS in adolescence is confounded by its overlapping features with normal pubertal changes. Three sets of diagnostic criteria have been developed, but a plethora of special considerations have been published [1,2,3]. The National Institutes of Health (NIH) criteria include chronic anovulation and hyperandrogenism, whereas the Rotterdam Consensus by the European Society of Human Reproduction and Embryology (ESHRE)/American Society for Reproductive Medicine (ASRM) broaden the PCOS criteria by including patients with anovulation and ovarian polycystic morphology without hyperandrogenism or with hyperandrogenism and polycystic morphology without anovulation [4,5]. The Androgen Excess and Polycystic Ovary Syndrome (AE-PCOS) Society united the criterion of ovarian polycystic morphology with that of anovulation under the term of ovarian dysfunction, and proposed the combination of either with hyperandrogenism to support the diagnosis of PCOS [6].

The clinical presentation of PCOS in adolescence shares common features with its presentation in adulthood, including the adverse metabolic profile [7]. PCOS is considered the ovarian equivalent of metabolic syndrome (MetS). The presence of adiposity and dependent or independent distorted insulin sensitivity are proposed as mediators [8].

Non-alcoholic fatty liver disease (NAFLD) or metabolic dysfunction-associated fatty liver disease (MAFLD) is the hepatic component of MetS. The former is defined as accumulation of liver fat more than 5% of the hepatic tissue [9]. It used to be a histological diagnosis of exclusion in the absence of alcohol abuse [10]. Recently, new criteria that combine hepatic steatosis with metabolic parameters have been proposed for the diagnosis of MAFLD [11]. The disease includes a spectrum of histological changes from relatively benign hepatic steatosis to fibrosis and carcinoma. Even in pediatrics, scientific societies have recognized the emerging epidemic and raise awareness for prevention and early diagnosis at a young age. Currently, the prevalence of NAFLD in children ranges between 7.6–9.6%, and quadruples in obese children [12]. Girls are less affected than boys (6.3% vs. 9%) [13]. The North American Society for Pediatric Gastroenterology, Hepatology and Nutrition (NASPGHAN) clinical practice guideline (2017) recommends persistent elevated serum alanine aminotransferase (ALT) levels as the best screening tool for children, whereas adult screening is straightforward with the use of liver enzymes or ultrasound alone or in combination [10,14].

Although the association between NAFLD and PCOS has been widely studied in adults, liver manifestations in adolescents with PCOS are largely unaddressed in the current literature. There are only seven original studies examining hepatic steatosis exclusively in adolescent patients [15,16,17,18,19,20,21,22,23]. Hepatic fibrosis has been assessed only in one study of PCOS patients, which included an adolescent sample [24]. Regarding the Greek population, NAFLD has been studied only in two adult PCOS samples and one adolescent sample [21,25,26]. Acknowledging this gap in the literature as well as PCOS’ metabolic implications and its comparable pathogenesis with NAFLD, we aimed to assess NAFLD and its risk factors in adolescents with PCOS. We investigated the burden of both steatosis and fibrosis and their associations with insulin resistance (IR), hyperandrogenism, and other metabolic characteristics in adolescents with PCOS.

## 2. Materials and Methods

This prospective case–control study was conducted over a period of three years, from January 2017 to January 2020.

Adolescent females were recruited from two tertiary Departments of the School of Medicine, National and Kapodistrian University of Athens in Greece: (i) the Center for Adolescent Medicine and UNESCO Chair in Adolescent Health Care of the First Department of Pediatrics, at the Aghia Sophia Children’s Hospital, and (ii) the Division of Pediatric-Adolescent Gynecology and Reconstructive Surgery of the Second Department of Obstetrics and Gynecology, at the Aretaieio Hospital. The study was approved by Ethics Committees of the two hospitals, the Aghia Sophia Children’s Hospital (protocol number 29661/23-12-16) and the Aretaieio Hospital (protocol number 61/19-06-18). Adolescents and their parents or guardians were informed about the aims and the procedure of the study and signed a consent form.

The Rotterdam criteria were used to diagnose PCOS, taking into consideration the recommendations of the Pediatric Endocrine Society [1,5,27]. Eligible adolescents should be at least two years after menarche, with persistent menstrual disorders. Adolescents with other hyperandrogenic or genetic/metabolic disorders, infections, use of steatogenic medications, alcohol consumption, and malnutrition that could cause secondary hepatic steatosis were excluded [6,14].

Controls were adolescents who presented for annual health care visits at least two years after menarche. Controls had normal menses and no signs of hyperandrogenism or history of liver or other diagnosed metabolic disorder. Increased body weight was not regarded as an exclusion criterion in any group.

At enrollment, adolescents’ anthropometric data and medical history were recorded. All anthropometric measurements were obtained by the same physician. Measurements of weight (in kilograms), height (Ht) in meters, hip circumference (HC), and waist circumference (WC) in centimeters were used to calculate the following anthropometric indices: body mass index (BMI) by weight/Ht^2^, waist to height ratio (WHtR) by WC/Ht, and waist to hip ratio (WHR) by WC/HC. Clinical hyperandrogenism (acne and hirsutism) were assessed in all adolescents. The modified Ferriman–Gallwey score was used to measure hirsutism.

Hormonal parameters were examined to define biochemical hyperandrogenism and to exclude other causes of menstrual disturbances. The early follicular phase of the menstrual cycle was chosen for blood sampling, in the morning after overnight fasting. Hormones and biochemical parameters were measured at the Choremeio Research Laboratory of the First Department of Pediatrics, at the Aghia Sophia Children’s Hospital, with commercial kits. Biochemical hyperandrogenism was assessed by the free androgen index (FAI), which was calculated by the equation FAI = total testosterone/sex hormone binding globulin × 100%. Additionally, the following non-invasive indices were calculated to assess IR: homeostatic model assessment for insulin resistance (HOMA-IR), quantitative insulin sensitivity check index (QUICKI), Matsuda index (M-ISI) with oral glucose tolerance test (OGTT) measurements where available, and glucose to insulin ratio (G/I ratio) [28,29,30,31] (Table 1). The HOMA-IR cut-off of 2.32 proposed by Chissini et al. for pubertal girls was used to define insulin resistance in our adolescent sample [32].

Ovarian volume was assessed by abdominal or transvaginal (in sexually active adolescents) ultrasound with the GE Veluson S10 ultrasound system by an experienced gynecologist of the Second Department of Obstetrics and Gynecology at the Aretaieio Hospital.

Liver steatosis was assessed by ultrasonographic evaluation of liver parenchyma echogenicity compared to right kidney cortex and spleen. The result was categorized as zero, mild, moderate, or severe liver steatosis [33] by two experienced radiologists of the Radiology Department, School of Medicine, National and Kapodistrian University of Athens at the Aretaieio Hospital. Additionally, adolescents were screened for hepatic fibrosis with transient elastography (Fibroscan^®^ device) at the 2nd Department of Internal Medicine, National and Kapodistrian University of Athens, at the Hippokratio General Hospital. Hepatic stiffness was expressed in kPa, and the 95th percentile of the Tokuhara study, 7.9 kPa, was used as cut-off [34].

Indices used to assess liver steatosis included the following: fatty liver index (FLI), liver fat score (LFS), lipid accumulation product (LAP), hepatic steatosis index (HSI), alanine aminotransferase to triglycerides ratio (ALT/TG), Tomizawa index, PCOS hepatic steatosis index for obese individuals (PCOS-HS), and visceral adiposity index (VAI) [16,35,36,37,38,39]. Liver fibrosis was assessed by the FIB-4 index, NAFLD fibrosis score (NFS), AST to platelet ratio index (APRI), BARD score, and BAAT score [40,41,42,43,44] (Table 1). Cut-off values proposed by their creators were used for all the indices.

The diagnosis of MetS in our adolescent sample was based on the International Diabetes Federation (IDF) criteria and on ethnic WC percentiles for the definition of abdominal obesity [45,46]. Categorical data are presented as frequencies, and continuous data as mean ± standard deviation (SD) or median (interquartile range), depending on normality test results. Liver stiffness value from Fibroscan^®^ and all calculated indices were used both as a continuous variables and categorical values depending on their proposed cut-offs. Statistical analyses were performed with SPSS 26.0 (SPSS Inc. Chicago, IL, USA). Comparisons were performed using *t*-test, Mann–Whitney U test, and Chi-squared test. Correlations were performed with multiple linear regression, and *p* < 0.05 was considered statistically significant.

## 3. Results

**Figure 1:**
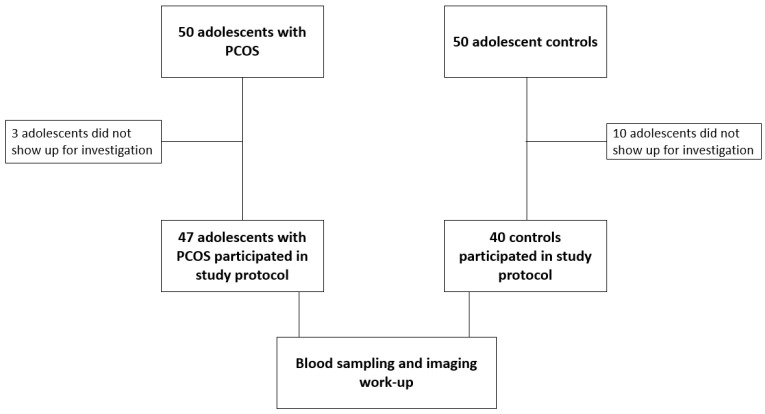
Flow chart of study participants.

A hundred Caucasian adolescent females were initially recruited in the study, thirteen of which did not show up for investigation, as shown in Figure 1.

A total of 87 adolescents aged 12.3–20.4 years (mean ± SD, 15.3 ± 1.6 years) were finally included in the study. The PCOS group included 47 adolescents with menstrual disorders and/or clinical hyperandrogenism and the control group included 40 age-matched female adolescents.

The demographic and anthropometric data, biochemical and hormonal parameters, and IR, steatosis, and fibrosis indices of the study groups are presented in Table 2. Adolescents of both groups had similar ages of menarche and levels of physical activity. Only 3.4% of all participants were diagnosed with MetS, all of them in the PCOS group. These three adolescents were also diagnosed with liver steatosis by ultrasound. No adolescent from the control group was subsequently (during work-up) diagnosed with PCOS.

### 3.1. PCOS Adolescents vs. Controls

Adolescents with PCOS had significantly higher BMI (*p* = 0.021) and higher HC (*p* = 0.011) than controls. WC and WHR were not significantly higher in PCOS adolescents (than controls), but in this group the prevalence of abdominal obesity was higher (17.4% vs. 2.5%, *p* = 0.024). Fasting glucose and IR markers HOMA-IR and QUICKI were more affected in adolescents with PCOS vs. controls (*p* = 0.004, *p* = 0.015, and *p* = 0.006, respectively). Adolescents with PCOS had lower high-density lipoprotein cholesterol (HDLc) (*p* = 0.039) and higher alanine aminotransferase (ALT) and gamma-glutamyl transferase (GGT) levels (*p* = 0.011 and *p* = 0.009, respectively) than controls.

By ultrasound, hepatic steatosis was found in 13.5% of all adolescents, in significantly (*p* = 0.046) more adolescents with PCOS (22.7%) than controls (6.1%). Regarding hepatic steatosis indices, FLI values were significantly higher in the PCOS (vs. control) group (*p* = 0.007). LFS score indicative of NAFLD presented in 17.5% of the whole sample, 24.4% in the PCOS group vs. 8.57% in the control group, with significantly higher median LFS score in adolescents with PCOS than in controls (*p* = 0.019). NAFLD was diagnosed by HSI in 29.1% of the whole sample, 40.9% in adolescents with PCOS vs. 14.2% in controls. The ALT to triglycerides ratio was positive for steatosis in 7.6% of the whole sample, 11.1% of those with PCOS, and 2.9% of controls, again with significantly different values between the two groups (*p* = 0.009). The prevalence of NAFLD diagnosed by the Tomizawa index was significantly higher in PCOS adolescents than controls. VAI was positive for steatosis in 6.6% of the PCOS group only, however, VAI as well as LAP scores did not differ significantly between PCOS adolescents and controls. PCOS-HS was dramatically high in both PCOS and control groups (45.2% and 50%, respectively), and was not used further in statistical analysis (Table 2).

Hepatic fibrosis as assessed by Fibroscan^®^ and calculated indices (FIB-4, NFS, BARD score, BAAT score, and APRI score) did not differ significantly between the PCOS and control groups. Fibroscan^®^ values indicative of hepatic fibrosis (≥7.9 kPa) were found in only six adolescents, three of each group. Pathological BAAT and APRI scores were found in only one and two adolescents, respectively. However, a BARD score ≥2 was reported in 94.7% of the whole sample. This was due to the high percentage of adolescents with an AST to ALT ratio ≥0.8 and BMI > 28 kg/m^2^, which are components of the score.

### 3.2. PCOS vs. Controls: BMI Stratification

To identify the influence of adiposity on PCOS-related hepatic steatosis, the study sample was stratified by BMI. In adolescents with BMI < 25 kg/m^2^, there were no significant differences between PCOS adolescents and controls regarding fatty liver diagnosis by scores or by ultrasound. On the contrary, among overweight/obese adolescents with BMI ≥ 25 kg/m^2^, those with PCOS had significantly more liver steatosis diagnoses by indices (but not by ultrasound) than their BMI-matched controls.

In both groups, overweight/obese adolescents had significantly worse anthropometric data, worse IR indices, and higher liver steatosis scores than those with BMI < 25 kg/m^2^. FAI, SBP, ALT, and GGT levels were also higher in overweight/obese adolescents with PCOS than those with BMI < 25 kg/m^2^, however, such significant differences were not observed in the control group (Table 3).

### 3.3. PCOS Adolescents: Low vs. High FAI

Since hyperandrogenism is an innate feature of PCOS, the PCOS group was stratified by the FAI cut-off of 5.26 (mean FAI of the PCOS group). Liver steatosis was worse in PCOS adolescents with worse hyperandrogenism than milder cases, as the LAP and HSI indices were significantly worse in those adolescents with high (vs. low) FAI (24.07 ± 15.06 vs. 13.49 ± 9.438, *p* = 0.08, and 39.88 ± 8.32 vs. 33.64 ± 5.709, *p* = 0.021, respectively).

### 3.4. PCOS Adolescents: Presence vs. Absence of Hepatic Steatosis

The PCOS group was stratified by the HSI cut-off of 36, as HSI > 36 is used to diagnose hepatic steatosis [38]. BMI, WC, HC, WHtR, and WHR were significantly higher in PCOS adolescents in the presence (vs. absence) of steatosis. Adolescents with PCOS in the presence (vs. absence) of steatosis also had significantly higher SBP and FAI, worse lipid profiles, and worse fasting indices of IR, namely HOMA-IR, QUICKI, and G/I ratio. Hepatic stiffness assessed by Fibroscan^®^ was more unfavorable in PCOS adolescents with steatosis than in PCOS adolescents without steatosis (*p* = 0.009) (Table 4).

### 3.5. PCOS Adolescents: Multiple Regression for NAFLD

After performing multiple regression models in the PCOS group, HOMA-IR was found to predict NAFLD diagnosis by the LFS index (β = 0.709, *p* = 0.002), but not FAI or testosterone levels. Other variables, such as BMI, were futile to be used as independent variables in regression models, since they were used to calculate the indices.

## 4. Discussion

The association between PCOS with NAFLD was described in 2005 by Brown et al., who reported a case of a 24-year-old woman with PCOS, elevated liver enzymes, and severe steatohepatitis in liver biopsy. Her clinical presentation improved significantly with weight loss [47].

Different diagnostic methods create great heterogeneity between studies, both in adult and in adolescent populations. Barfield et al. used elevated liver enzymes and reported a prevalence of NAFLD in obese PCOS adolescents of 15.4%, whereas Michaliszyn et al., with the use of CT abdominal scan reported a prevalence of 6.7% [19,20]. As already mentioned, ΝASPGHAN proposes the use of persistently elevated ALT levels (twice the upper normal limit of 22 U/L for girls) as a screening method. In our sample, 15% had ALT > 22 U/L but only one adolescent had ALT > 44 U/L. Since our study protocol did not include repeated blood sampling, and due to very low specificity, we did not use this method [14,48]. Imaging methods such as ultrasound also have problematic diagnostic accuracy. According to NASPGHAN, ultrasound in childhood has low sensitivity and specificity and inadequate quantification when liver steatosis involves <33% of hepatocytes [49]. A plethora of non-invasive indices have been created over the years to screen the general population or special groups for steatosis or fibrosis. They utilize history and anthropometric or biochemical data and have acceptable sensitivity and specificity [16,35,36,37,38,39,40,41,42,43,44,50,51,52]. Polyzos et al. used a variety of NAFLD indices to assess differences between adults with PCOS and controls, and reported all steatosis and fibrosis scores significantly higher in PCOS patients than controls, especially in those with concurrent MetS [25].

We also found significantly higher scores of hepatic steatosis by most of the indices used in PCOS adolescents compared to controls. Specifically, ultrasound echogenicity, LFS, FLI, HSI, and ALT/TG ratio were significantly higher in PCOS adolescents than controls. On the contrary, fibrosis scores did not differ significantly between PCOS adolescents and controls. The young age of our sample may explain this finding, since fibrosis evolves over time and is not common in adolescence.

Baranova et al. systematically reviewed all known genes that associate PCOS with MetS and NAFLD. Their web is formed by androgen biosynthesis and signaling pathways, adiposity, and adipokine and cytokine genes, in combination with insulin signaling pathways [53]. These four pillars, adiposity, hyperandrogenism, IR, and inflammation, prevail in PCOS epidemiological studies.

A meta-analysis of 17 studies reported a 2.5-fold increase in the risk of NAFLD for PCOS patients. Additionally, testosterone levels and FAI were reported as independent predictors for NAFLD after controlling for metabolic parameters such as age, BMI, triglyceride levels, and IR [54]. The results of another meta-analysis were consonant, reporting ORs of 2.25 and up to 3.01 for obese and 2.07 for lean patients. In this study, hyperandrogenism was recognized as the main determinant, as only patients with hyperandrogenism had higher risk than controls. In our study, liver steatosis was worse in PCOS adolescents with high (vs. low) FAI.

Adolescents with PCOS and NAFLD had significantly higher BMI, waist circumference, FAI, and IR than PCOS patients without liver involvement. Similar results were found in the study of Ayonrinde et al., who compared adolescent girls with PCOS with adolescent boys and found comparable metabolic profiles [15].

IR appears as the key intermediary between PCOS and NAFLD. IR is involved in the pathogenesis of both disorders. It is well known that insulin sensitivity in PCOS is affected not only in women with overweight or obesity, but also in those with normal weight [55]. Even in adolescents, PCOS is a risk factor for impaired glucose tolerance [56,57]. Insulin resistance in the skeletal muscle in PCOS is mediated through impaired IRS-1 expression and phosphorylation, post-receptor signaling defect, and abnormal GLUT4 translocation [53,58]. In our sample, adolescents with PCOS and NAFLD had worse insulin sensitivity indices (HOMA-IR, QUICKI, and G/I ratio) than adolescents with PCOS alone. Furthermore, only HOMA-IR was found to predict NAFLD diagnosis by the LFS index. To confirm the predictive role of insulin resistance using HOMA-IR in PCOS steatosis, more studies in larger populations and different ethnic backgrounds are warranted. Whether nutritional or medical interventions to decrease insulin resistance in adolescents with high BMI, PCOS, and NAFLD will lead to better prognosis remains a valid question for future research.

Studies assessing NAFLD in adolescents with PCOS are scarce in the literature. Beyond Ayonrinde et al., as mentioned before, Cree-Green et al. found that only 14% of obese girls without PCOS had NAFLD, whereas 3-fold obese PCOS patients had NAFLD [15,18]. The same team created in 2019 a new tool to diagnose NAFLD, particularly in obese adolescents, with a sensitivity of 82% and specificity 69% for the cut-off value of 0.44. We used this PCOS-HS index in both lean and overweight/obese adolescents, but a surprisingly high prevalence was found with no significant differences between PCOS adolescents and controls.

In the most recent study by Won et al., a higher prevalence of NAFLD was diagnosed in adolescents than in adults (11.8% vs. 8.1%). However, this difference could be attributed to the different criteria used for the PCOS diagnosis in the two age groups [24]. Using hyperandrogenism as a prerequisite for PCOS diagnosis creates a more homogeneously metabolically affected sample. In our study, PCOS patients with more serious hyperandrogenism presented worse liver steatosis profiles than milder cases. As for hepatic fibrosis, measured by Fibroscan^®^, our PCOS sample demonstrated a mean value of 6.25 ± 1.55 kPa, very similar to the 6.6 (±2.1) kPa that was found by Won et.al.

Study limitations include the relatively small sample size, the lack of longitudinal follow-up, and the absence of histologic confirmation for NAFLD; however, non-invasive assessment is usually recommended in youth. There is controversy in the literature regarding the diagnosis of adolescent PCOS. In this study, the Rotterdam criteria were used, whereas pediatric endocrine and other societies’ recommendations were also taken into consideration to minimize false-positive diagnoses of PCOS. Regarding the diagnosis of NAFLD, the reference population of the calculated indices are adults. Indices for the diagnosis of adolescent NAFLD have not been developed, except from PCOS-HS which led to a surprisingly high diagnosis in our sample. Nevertheless, to our knowledge this is the first study to assess hepatic steatosis and fibrosis of adolescents with PCOS in Greece. Additionally, our study included both lean and overweight/obese adolescents, in contrast to other studies which included only overweight/obese individuals. Finally, both imaging modalities and calculated indices were used to assess hepatic involvement.

## 5. Conclusions

PCOS has been proven true to its characterization as a syndrome since more and more disorders have been related over the years to menstrual irregularities and androgen hyperproduction. The metabolic burden, known for a long time now, includes the hepatic manifestation of NAFLD/MAFLD. In our adolescent sample, NAFLD diagnosis, both by ultrasound and non-invasive steatosis indices, was more prevalent in adolescents with PCOS than controls. The latter was not apparent for the diagnosis of hepatic fibrosis. The HOMA score for IR was predictive of steatosis. PCOS adolescents with hepatic steatosis presented with higher BMI and abdominal adiposity, higher SBP, worse insulin sensitivity, and worse hyperandrogenism than PCOS adolescents without steatosis. Despite its limitations, our study suggests a high suspicion for hepatic steatosis in PCOS patients with higher BMI, IR, and hyperandrogenism even in adolescence. Larger studies are needed to verify these findings.

## Figures and Tables

**Table 1:** Insulin resistance, steatosis, and fibrosis indices used in the study.

Homeostatic model assessment for insulin resistance (HOMA-IR)	(HOMA-IR = fasting glucose (mg/dL) × insulin (μU/mL)/405)	
Quantitative insulin sensitivity check index (QUICKI)	QUICKI = 1/[log(fasting insulin μU/mL) + log(fasting glucose mg/dL)]	
Matsuda index (M-ISI)	M-ISI = 10,000/(glucose_0_ mmol/dL × insulin_0_ mU/L × glucose mean mmol/dL × insulin-mean mU/L)1/2	
Glucose to insulin ratio (G/I ratio)	G/I ratio = fasting glucose/fasting insulin	
Fatty liver index (FLI)	FLI = (e0.953 × loge(TG) + 0.139 × BMI + 0.718 ∗ loge(GGT) + 0.053 × WC − 15.745)/(1 + e0.953 × loge(TG) + 0.319 × BMI + 0.718 × loge(GGT) + 0.053 × WC − 15.745) × 100	
Liver fat score (LFS)	LFS = −2.89 + 1.18 × MetS (yes = 1, no = 0) + 0.45 × T2DM (yes = 2, no = 0) + 0.15 × fS-insulin (mU/L) + 0.04 × fS-AST (U/L) − 0.94 × AST/ALT	
Lipid accumulation product (LAP)	LAP = (WC [cm] − 58) × (TG [mmol/L])	
Hepatic steatosis index (HSI)	HIS = 8 × ALT/AST ratio +BMI(+2 if T2DM, +2 if female)	
ALT/TG ratio	ALT/TG	
Tomizawa index	positive if ALT > 19 IU/L and TG > 101 mg/dL	
PCOS hepatic steatosis index for obese individuals (PCOS-HS)	PCOS-HS = 1/1 + [exp (−(26.01 + (−0.3761 × BMI percentile + 0.05781 × WC (cm) + 0.0448 × HOMA IR + 0.00095519 × HDLc (mmol/L) + 0.00005892 × TG (mmol/L) + 0.0964 × ALT (IU/L) + 0.001548 × free testosterone (nmol/L) − 0.06806 × SHBG (nmol/L))]	
Visceral adiposity index (VAI)	VAI = [WC/(36.58 + 1.88 × BMI)] × TG/0.81 × 1.52/HDLc	
FIB-4 index	FIB-4 = age (years) × AST(U/L)/[PLT(109/L) × ALT1/2 (U/L)]	
NAFLD fibrosis score (NFS)	NFS = −1.6750 + 0.037 × age (years) + 0.094 × BMI (kg/m^2^) + 1.13 × impaired fasting glucose/T2DM (yes = 1, no = 0) + 0.99 × AST/ALT ratio − 0.013 × PLT (109/L) − 0.66 × albumin (g/dL)	
AST to platelet ratio index (APRI)	APRI = (AST/upper limit of AST × 100)/PLT (×109/L)	
BARD score	BARD = sum of points, BMI ≥ 28 = 1 point, AST/ALT ≥ 0.8 = 2 points, T2DM = 1 point	
BAAT score	BAAT = sum of points, BMI ≥ 28 = 1 point, <28 = 0, age ≥ 50 = 1 point, <50 = 0, ALT ≥ 2 × upper normal = 1 point, <2 upper normal = 0, TG ≥ 1.7 mmol/L = 1, <1.7 = 0	

Abbreviations: BMI, body mass index; WC, waist circumference; MetS, metabolic syndrome; T2DM, type 2 diabetes mellitus; HDLc, high-density lipoprotein cholesterol; AST, aspartate aminotransferase; ALT, alanine aminotransferase; GGT, gamma-glutamyl transferase; TG, triglycerides; PLT, platelet number; PCOS, polycystic ovary syndrome; SHBG, sex hormone binding globulin; NAFLD, non-alcoholic fatty liver disease.

**Table 2:** Demographic and anthropometric data, biochemical and hormonal parameters, and IR, steatosis, and fibrosis indices (as continuous variables) of PCOS and control groups.

Variables	PCOS Group (*n* = 47)	Control Group (*n* = 40)	*p*-Value
Age (years) ^§^	15.66 (2.49)	14.81 (2.00)	0.082
Menarche (years)	11.96 ± 1.41	11.68 ± 0.91	0.281
Physical activity (h/w) ^§^	1.00 (5.00)	2.00 (4.75)	0.797
BMI (kg/m^2^)	25.56 ± 5.28	23.14 ± 4.07	**0.021**
SBP (mmHg)	116.35 ± 10.44	113.00 ± 7.75	0.116
DBP (mmHg)	67.92 ± 10.35	66.30 ± 7.45	0.442
Waist circumference (cm)	76.36 ± 10.23	72.71 ± 7.90	0.071
Hip Circumference (cm)	102.54 ± 10.21	97.20 ± 8.57	**0.011**
Waist to height ratio	0.47 ± 0.07	0.46 ± 0.05	0.196
Waist to hip ratio	0.744 ± 0.058	0.749 ± 0.068	0.704
AST (U/L) ^§^	17.50 (6.00)	16.00 (5.00)	0.315
ALT (U/L) ^§^	14.00 (10.00)	13.00 (6.00)	**0.011**
GGT (U/L) ^§^	11.90 (8.00)	10.00 (5.00)	**0.009**
ALP (U/L) ^§^	74.00 (27.00)	82.00 (40.00)	0.388
TC (mg/dL) ^§^	148.50(28.00)	146.50 (43.15)	0.657
LDLc (mg/dL) ^§^	84.00 (32.50)	82.10 (34.70)	0.984
HDLc (mg/dL)	53.38 ± 12.54	59.19 ± 11.76	**0.039**
TG (mg/dL) ^§^	66.65 (41.00)	59.00 (32.30)	0.699
Lpa (nmol/L) ^§^	14.50 (40.70)	12.10 (15.25)	0.380
ApoB (mg/dL) ^§^	74.60 (21.00)	55.00 (32.00)	0.308
ApoA1 (mg/dL)	135.12 ± 19.11	141.83 ± 30.29	0.533
VitD (ng/mL)	26.13 ± 8.66	26.70 ± 7.76	0.790
Glu (mg/dL)	87.88 ± 7.48	83.37 ± 5.92	**0.004**
Insulin (μU/mL) ^§^	11.80 (7.80)	9.60 (6.50)	0.066
Ovarian volume (cm^3^)	11.45 ± 3.71	5.52 ± 2.22	**<0.001**
G/I ratio ^§^	7.37 (5.15)	8.58 (6.95)	0.090
QUICKI	0.33 ± 0.02	0.35 ± 0.03	**0.006**
HOMA-IR ^§^	2.45 (1.63)	1.88 (1.24)	**0.015**
LFS ^§^	−1.56 (1.32)	−2.07 (1.21)	**0.019**
FLI ^§^	2.78 (22.82)	0.44 (2.82)	**0.007**
HIS ^§^	35.46 (10.73)	30.98 (6.33)	**0.004**
ALT/TG ratio ^§^	3.73 (2.49)	3.12 (1.67)	**0.009**
Tomizawa index	8.5%	0%	**0.05**
VAI ^§^	0.92 (0.55)	0.75 (0.65)	0.239
LAP	16.62 ± 12.00	12.76 ± 8.05	0.078
PCOS-HS	0.48 ± 0.44	0.48 ± 0.46	0.998
FIB-4 ^§^	0.24 (0.08)	0.24 (0.11)	0.178
NFS	−4.53 ± 1.03	−4.16 ± 1.31	0.411
APRI ^§^	0.20 (0.09)	0.18 (0.10)	0.957
BAAT	2.1%	0%	0.353
BARD	92.5%	97.2%	0.357
Fibroscan^®^ stiffness (kPa)	6.25 ± 1.55	6.87 ± 3.61	0.570

Abbreviations: IR, insulin resistance; h, hour; w, week; BMI, body mass index; SBP, systolic blood pressure; DBP, diastolic blood pressure; TC, total cholesterol; TG, triglycerides; HDLc, high-density lipoprotein cholesterol; AST, aspartate aminotransferase; ALT, alanine aminotransferase; GGT, gamma-glutamyl transferase; ALP, alkaline phosphatase; HDLc, high-density lipoprotein cholesterol; LDL, low-density lipoprotein; Lpa, lipoprotein (a); Apo A1, Apolipoprotein A1; ApoB, Apolipoprotein B; VitD, 25-OH-vitamin D; Glu, fasting glucose; QUICKI, quantitative insulin sensitivity check index; HOMA-IR, homeostatic model assessment for insulin resistance; G/I ratio, glucose to insulin ratio; LFS, liver fat score; FLI, fatty liver index; HSI, hepatic steatosis index; VAI, visceral adiposity index; LAP, lipid accumulation product; PCOS-HS, PCOS hepatic steatosis index; PCOS, polycystic ovary syndrome; FIB-4, fibrosis score 4; NFS, NAFLD fibrosis score; NAFLD, non-alcoholic fatty liver disease; APRI, AST to platelet ratio index. Values refer to mean ± standard deviation and *t*-test or ^§^ median (interquartile range) and Mann–Whitney U. Bold indicates statistically significant differences.

**Table 3:** Anthropometric, biochemical/hormonal parameters, IR, and liver steatosis indices of PCOS and control groups stratified by BMI.

Variables	Control Group BMI < 25 kg/m^2^ (*n* = 28)	Control Group BMI ≥ 25 kg/m^2^ (*n* = 12)	*p*-Value	PCOS Group BMI < 25 kg/m^2^ (*n* = 24)	PCOS Group BMI ≥ 25 kg/m^2^ (*n* = 23)	*p*-Value
SBP (mmHg)	111.20 ± 6.72	116.75 ± 8.66	0.067	111.28 ± 8.64	119.48 ± 9.09	**0.007**
WC (cm)	67.41 ± 8.19	69.04 ± 7.17	**<0.001**	68.61 ± 4.43	83.22 ± 8.89	**<0.001**
HC (cm) ^§^	94.35 ± 6.55	103.83 ± 9.30	**<0.001**	96.00 (7.00)	109.00 (11.00)	**<0.001**
WHtR	0.43 ± 0.04	0.51 ± 0.02	**<0.001**	0.42 ± 0.03	0.52 ± 0.05	**<0.001**
GGT (U/L) ^§^	9 (5)	10 (6.5)	0.602	10.00 (4.50)	15.00 (9.00)	**0.014**
FAI ^§^	1.89 (2.13)	2.4 (1.3)	0.384	2.76 (2.14)	4.92 (4.68)	**0.009**
QUICKI	0.35 ± 0.02	0.33 ± 0.02	**0.002**	0.34 ± 0.02	0.32 ± 0.02	**0.012**
HOMA-IR ^§^	1.58 (0.94)	2.56 (2.09)	**0.006**	2.01 (1.04)	2.98 (2.48)	**0.012**
G/I ratio	11.67 ± 5.66	6.94 ± 2.42	**0.002**	9.69 ± 3.41	6.56 ± 2.52	**0.002**
ALT (U/L) ^§^	13 (6)	11 (5.25)	0.439	13.00 (7.00)	20.00 (11.00)	**0.040**
LFS ^§^	−2.38 (1.32)	−1.56 (1.20)	**0.011**	−1.97 (1.03)	−1.20 (1.67)	**0.007**
FLI ^§^	0.36 (0.37)	4.76 (3.62)	**<0.001**	0.37 (0.63)	19.71 (22.92)	**<0.001**
LAP	8.98 ± 5.43	21.57 ± 2.42	**<0.001**	8.07 ± 4.38	24.55 ± 11.41	**<0.001**
HSI ^§^	29.11 (4.47)	35.86 (3.62)	**<0.001**	29.24 (2.33)	40.10 (6.40)	**<0.001**

Abbreviations: BMI, body mass index; IR, insulin resistance; PCOS, polycystic ovary syndrome; SBP, systolic blood pressure; WC, waist circumference; HC, hip circumference; WHtR, waist to height ratio; GGT, gamma-glutamyl transferase; FAI, free androgen index; G/I ratio, glucose to insulin ratio; QUICKI, quantitative insulin sensitivity check index; HOMA-IR, homeostatic model assessment for insulin resistance; ALT, alanine aminotransferase; LFS, liver fat score; FLI, fatty liver index; LAP, lipid accumulation product; HSI, hepatic steatosis index. Values refer to mean ± standard deviation and *t*-test or ^§^ median (interquartile range) and Mann–Whitney U. Bold indicates statistically significant differences.

**Table 4:** Significant differences in anthropometric, biochemical/hormonal parameters, IR, and liver fibrosis indices between PCOS adolescents with or without hepatic steatosis.

	HSI ≤ 36 (*n* = 26)	HSI > 36 (*n* = 18)	*p*-Value
BMI (kg/m^2^)	22.33 ± 3.09	30.69 ± 3.98	**<0.001**
Waist circumference (cm)	70.80 ± 5.53	85.31 ± 9.22	**<0.001**
Hip circumference (cm)	96.82 ± 7.06	111.03 ± 8.86	**<0.001**
Waist to height ratio	0.44 ± 0.04	0.53 ± 0.06	**<0.001**
Waist to hip ratio	0.73 ± 0.45	0.77 ± 0.06	**0.030**
SBP (mmHg)	113.57 ± 8.93	121.06 ± 11.05	**0.025**
FAI ^§^	3.05 (3.48)	5.31 (5.69)	**0.034**
Insulin (μU/mL) ^§^	9.40 (3.75)	15.00 (6.40)	**0.001**
QUICKI	0.34 ± 0.02	0.32 ± 0.02	**0.002**
HOMA-IR ^§^	2.02 (0.89)	3.16 (2.03)	**0.002**
G/I ratio	9.34 ± 3.33	5.88 ± 2.42	**0.001**
Total cholesterol (mg/dL)	143.12 ± 18.44	159.27 ± 29.49	**0.031**
LDLc (mg/dL)	77.56 ± 20.21	94.07 ± 29.08	**0.031**
Triglycerides ^§^ (mg/dL)	51.50 (30.90)	75.00 (31.00)	**0.019**
Fibroscan^®^ stiffness (kPa)	5.30 ± 1.30	7.37 ± 0.99	**0.009**

Abbreviations: IR, insulin resistance; PCOS, polycystic ovary syndrome; HSI, hepatic steatosis index; BMI, body mass index; SBP, systolic blood pressure; FAI, free androgen index; QUICKI, quantitative insulin sensitivity check index; HOMA-IR, homeostatic model assessment for insulin resistance; G/I ratio, glucose to insulin ratio; LDLc, low-density lipoprotein cholesterol. Values refer to mean ± standard deviation and *t*-test or ^§^ median (interquartile range) and Mann–Whitney U. Bold indicate statistically significant differences.

## Data Availability

Research data are available upon request.
